# Multiparasitism by myxozoans in *Pygocentrus nattereri* (Characiformes: Serrasalmidae) from Sacaizal Lake, in the municipality of Pracuúba, state of Amapá, Brazil

**DOI:** 10.1590/S1984-29612025020

**Published:** 2025-04-14

**Authors:** Nayana Moraes de Sena, Jhonata Eduard, Camila Maria Barbosa Pereira, José Ledamir Sindeaux, Michele Velasco

**Affiliations:** 1 Laboratório de Integração Morfo-Molecular e Tecnologias – LIMT, Instituto de Saúde e Produção Animal – ISPA, Universidade Federal Rural da Amazônia – UFRA, Belém, PA, Brasil; 2 Programa de Pós-graduação em Saúde e Produção Animal na Amazônia – PPGSPAA, Universidade Federal Rural da Amazônia – UFRA, Belém, PA, Brasil; 3 Programa de Pós-graduação em Biologia de Agentes Infecciosos e Parasitários – BAIP, Universidade Federal do Pará – UFPA, Belém, PA, Brasil; 4 Programa de Pós-graduação em Biodiversidade e Biotecnologia – PPG-BIONORTE, Universidade Federal do Pará – UFPA, Belém, PA, Brasil; 5 Programa de Pós-graduação em Reprodução Animal na Amazônia – REPROAMAZON, Universidade Federal Rural da Amazônia – UFRA, Belém, PA, Brasil

**Keywords:** Amazon, fish, Henneguya, Myxobolus, myxozoans, piranha, Amazônia, peixe, Henneguya, Myxobolus, mixozoários, piranha

## Abstract

The present study described the morphological characteristics of myxospores of cnidarians belonging to the class Myxozoa at different infection sites in *Pygocentrus nattereri* Kner 1858 (Characiformes: Serrasalmidae), found in Lake Sacaizal, in the municipality of Pracuúba, state of Amapá, Brazil. In 44% of the specimens analyzed, myxospore gill filaments of the genus *Henneguya* were observed with a pyriform sporal body, presenting long caudal projections internally, with two polar capsules. In some filaments, branchial arches, and fin cysts (with a prevalence of 22%) pyriform *Myxobolus* myxospores were observed, which were larger than those found in the caudal kidneys, where the myxospores had an elliptical shape, demonstrating the presence of two morphotypes in the same host. Thus, these morphological data contribute to the diversity of myxozoans in Amazonian fish, particularly *P. nattereri* in their natural environment, with the first occurrence of these parasite genera in this host.

## Resumo

O presente estudo descreveu as características morfológicas de mixósporos de cnidários pertencentes à classe Myxozoa em diferentes sítios de infecção em *Pygocentrus nattereri* Kner 1858 (Characiformes: Serrasalmidae), encontrado no Lago Sacaizal, no município de Pracuúba, estado do Amapá, Brasil. Em 44% dos espécimes analisados ​​foram observados nos filamentos branquiais mixósporos do gênero *Henneguya* com corpo esporal piriforme, apresentando projeções caudais longas internamente, com duas cápsulas polares. Em alguns filamentos, arcos branquiais e cistos de nadadeiras (com prevalência de 22%) foram observados mixósporos piriformes do gênero *Myxobolus*, que eram maiores que os encontrados nos rins caudais, onde os mixósporos apresentavam formato elíptico, demonstrando a presença de dois morfotipos no mesmo hospedeiro. Assim, esses dados morfológicos contribuem para a diversidade de mixozoários em peixes amazônicos, particularmente em *P. nattereri* em seu ambiente natural, com a primeira ocorrência desses gêneros de parasitos neste hospedeiro.

## Introduction

Myxozoans are metazoan parasites that infect flatworms, reptiles, amphibians, fish, both marine and freshwater, and particularly teleost fish species (Kent et al., 2001; Okamura et al., 2015; Eiras et al., 2023; Adriano & Eiras, 2024). Most myxozoans have an indirect life cycle involving a definitive host, such as an oligochaete, polychaeta, or bryozoan, and an intermediate host, in this case fish (Yokoyama et al., 2012). In fish, myxosporidia are mainly found in the gills, epithelial tissue, fins, skeletal muscles, gallbladder, and other vital organs (Eiras et al., 2023).

The class Myxozoa Grassé, 1970 comprises 64 genera (Fiala et al., 2015), where the genera *Myxobolus* Bütschli, 1882 and *Henneguya* Thélohan, 1892 (Myxobolidae) have the largest number of species, together comprising almost half of the overall diversity of the known species in this class (Liu et al., 2019). Considering their pathogenic potential, these parasites can affect fisheries and aquaculture production (Moran et al., 1999), causing damage to the cells of the tissue they infect, which can adversely affect the health of the hosts (Sterud et al., 2007).

*Myxobolus cerebralis* Hofer, 1903 has been shown to be a significant factor in the decline of wild host fish stocks (Feist & Longshaw, 2006), causing salmonid whirling disease. *Henneguya* species exhibit severe hyperplasia and fusion of secondary gill filaments, congestion of blood vessels that constitute the gill arches, and deformation of the lamellar structure, including capillary compression, edema, and thickening of the epithelial surface (Barassa et al., 2003; Eiras et al., 2008; Sales et al., 2020).

Some species of Myxozoa are highly pathogenic and cause severe diseases in fish, such as *Kudoa thyrsites* Gilchrist, 1923 (Langdon, 1991) and *M. cerebralis* (Hoffman, 1990). However, species with low pathogenicity are present that cause small-scale pathologies and can only be detected through microscopic and histological analyses (Barassa et al., 2003; Velasco et al., 2016; Figueredo et al., 2020; Sales et al., 2020).

Fish of genus *Pygocentrus* belongs to the order Characiformes and family Serrasalmidae. This genus comprises four species that are popularly known as piranhas and are widely distributed in river basins in South America (Nelson et al., 2016). *Pygocentrus nattereri* Kner, 1858, is pelagic, has diurnal and nocturnal habits, is carnivorous, ingests mainly fish, and consumes insects, crustaceans, and parts of mammals (bats and capybarafur) and plant materials (Soares et al., 2008; Ferreira et al., 2014). For *P. nattereri*, only two species of Myxozoa, *Ellipsomyxa arariensis* Silva, Matos, Lima, Furtado, Hamoy & Matos, 2018 (Silva et al., 2018), and *Myxobolus dermatoulcerans* Stilwell, Stilwell, Camus, Ware, Rosser & Griffin, 2020 (Stilwell et al., 2020) are known.

The aims of this study were to describe the morphological aspects of *Henneguya* sp. parasitizing the gill filament and two morphotypes of *Myxobolus* sp. found in the gill arch and filament, caudal fin, and kidney of *P. nattereri* in the Amazon.

## Material and Methods

### Specimen collection and study area

**Figure 1:**
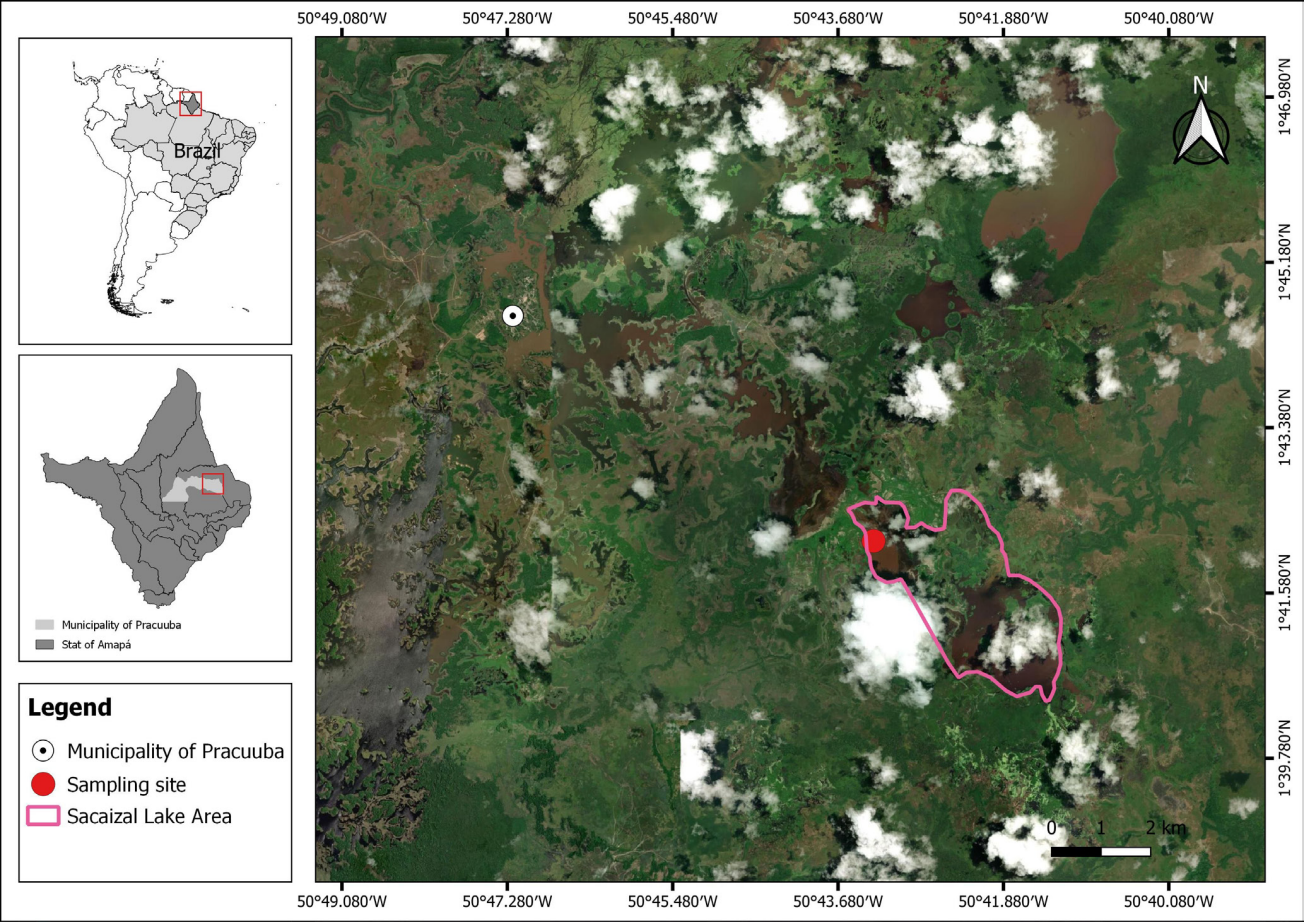
Geographic location map of the collection site of *Pygocentrus nattereri* in the municipality of Pracuúba, state of Amapá, Brazil.

Eighteen specimens of *P. nattereri* (Biodiversity Authorization and Information System - SISBIO/ICMBio License n° 88196-1) were collected using cast nets in Lake Sacaizal, located in the municipality of Pracuúba (1°42'8.79"N 50°43'17.56"W), state of Amapá (Figure 1). Fish were purchased from artisanal fishermen, stored in isothermal boxes with ice, and transported to the Laboratory of Morpho-Molecular Integration and Technologies (LIMT) at the Federal Rural University of the Amazon (UFRA) in Belém, state of Pará (Brazil, where biometric data were measured and necropsy was performed (Animal Use Ethics Committee CEUA n° 7218270723/ID 000609). Using a stereoscope, parasites were detected across the entire body surface in tissues and organs. Tissue fragments were removed and observed under a light microscope to confirm the presence of Myxozoan parasites.

### Myxospore morphometry

The myxospores found were measured in micrometers (µm) using the ImageJ program version 1.46r, and several morphometric parameters were obtained from the *Henneguya* myxospores, according to the taxonomic description of myxozoans recommended by Lom & Arthur (1989), such as total size (TT), tail length (CC), myxospore length (CE), myxospore width (LE), polar capsule length (CCP), polar capsule width (LCP), number of turns of the polar filament (NVFP), and parameters of *Myxobolus* sp*.*: myxospore length.

These data were compared with information available for other species described in previous studies (Tables 1 and 2) using principal component analysis (PCA), using rotation varimax, performed using PAST software version 4.03 for statistical analysis (Hammer et al., 2001).

**Table 1:** Morphological data (µm) of *Henneguya* sp. in Amazonian fishes.

**SPECIES OF MYXOZOA**	**SPECIES OF HOSTS**	**SITE OF INFECTION**	**LOCALITIES**	**TT**	**MYXOSPORE BODY**		**CC**	**POLAR CAPSULE**		**NVFP**	**REFERENCES**
					**Length**	**Width**		**Length**	**Width**		
*Henneguya* sp.	*Pygocentrus nattereri*	Gill filament	State of Amapá, Brazil	32.4	12.9	4.5	19.0	6.6	1.2	14-15	Present study
*Henneguya paraensis*	*Cichla temensis*	Gill	State of Pará, Brazil	42.3	12.8	8.6	29.5	7.4	2.6	5-7	Velasco et al. (2016)
*Henneguya aequidens*	*Aequidens plagiozonatus*	Gill	State of Pará, Brazil	41	15	6	27	3	2	4-6	Videira et al. (2015)
*Henneguya sacacaensis*	*Satanoperca jurupari*	Gill	State of Amapá, Brazil	46.5	16.5	5.1	30	3.83	1.68	7-9	Ferreira et al. (2020)
*Henneguya rhamdia*	*Rhamdia quelen*	Gill	State of Pará, Brazil	50	13.1	5.2	36.9	4.7	1.1	10-11	Matos et al. (2005)
*Henneguya amazonica*	*Crenicichla lepidota*	Gill	State of Pará, Brazil	59.3	13.9	5.7	45.4	3.3	1.5	-	Rocha et al. (1992)
*Henneguya santarenensis*	*Phractocephalus hemioliopterus*	Gill	State of Pará, Brazil	31.9	10.8	4.3	21	4.6	1.4	15	Naldoni et al. (2018)

TT: Total size; CC: Tail length; NVFP: Number of turns of the polar filament.

**Table 2:** Morphometric data (µm) of *Myxobolus* sp. in the Amazonian fishes.

**SPECIES OF MYXOZOA**	**SPECIES OF HOSTS**	**SITE OF INFECTION**	**LOCALITIES**	**MYXOSPORE BODY**		**POLAR CAPSULE**		**NVFP**	**REFERENCES**
				**Length**	**Width**	**Length**	**Width**		
*Myxobolus* sp.1 (oval)	*Pygocentrus nattereri*	Gill filament, caudal fin, and kidney	State of Amapá, Brazil	**18.5**	**10**	**9.1**	**2.9**	**-**	**Present study**
*Myxobolus* sp.2 (drop)	*Pygocentrus nattereri*	Branchial arch and kidney	State of Amapá, Brazil	**12**	**7.5**	**8.4**	**1.9**	**-**	**Present study**
*Myxobolus* sp.3	*Metynnis lippincottianus*	Blood and kidney	State of Amapá, Brazil	17	6	15	2.4	16-18	Façanha et al. (2024)
*Myxobolus* sp.4	*Metynnis lippincottianus*	Blood and kidney	State of Amapá, Brazil	11	4.1	6.5	1.6	4-6	Façanha et al. (2024)
*Myxobolus* sp.5	*Metynnis hypsauchen*	Kidney	State of Pará, Brazil	12,5	7.3	6.2	2.8	8-10	Oliveira et al. (2020)
*Myxobolus maculatus*	*Metynnis maculatus*	Kidney	State of Pará, Brazil	21	8.9	12.7	3.2	14-15	Casal et al. (2002)
*Myxobolus tapajosi*	*Brachyplatystoma rousseauxii*	Gill filament	State of Pará, Brazil	15	10.7	5.8	3.0	6-7	Zatti et al. (2018)
*Myxobolus. niger*	*Corydoras melini*	Branchial arch	State of Amazonas, Brazil	11.3	6.8	5.0	2.0	6-7	Mathews et al. (2016)
*Myxobolus marajoensis*	*Rhamdia quelen*	Intestine	State of Pará, Brazil	10.9	5.1	5.3	1.6	-	Abrunhosa et al. (2017)
*Myxobolus metynnis*	*Metynnis argenteus*	Orbicularis region	State of Pará, Brazil	13.1	7.8	5.2	3.2	8-9	Casal et al. (2006)
*Myxobolus freitasi*	*Brachyhypopomus beebei*	Brain	State of Pará, Brazil	18.6	8.6	13.0	5.6	14-15	Sindeaux-Neto et al. (2021)

NVFP - Number of turns of the polar filament.

### Scanning Electron Microscopy (SEM)

Tissue fragments and myxospores were fixed in 5% glutaraldehyde buffered with 0.2 M sodium cacodylate at pH 7.2 for 3 h at 4 ºC. They were then washed in the same buffer for 2–4 h at 4 ºC, and post-fixed in 2% osmium tetroxide in the same buffer for 2 h at the same temperature, and dehydrated in an increasing series of ethanol. They were dried to the critical point and metallized with gold. Visualizations and photomicrographs were obtained using a Tescan Mira3 scanning electron microscope at the Scanning Electron Microscopy Laboratory of the Research Campus of the Museu Paraense Emílio Goeldi - MPEG.

## Results

**Figure 2:**
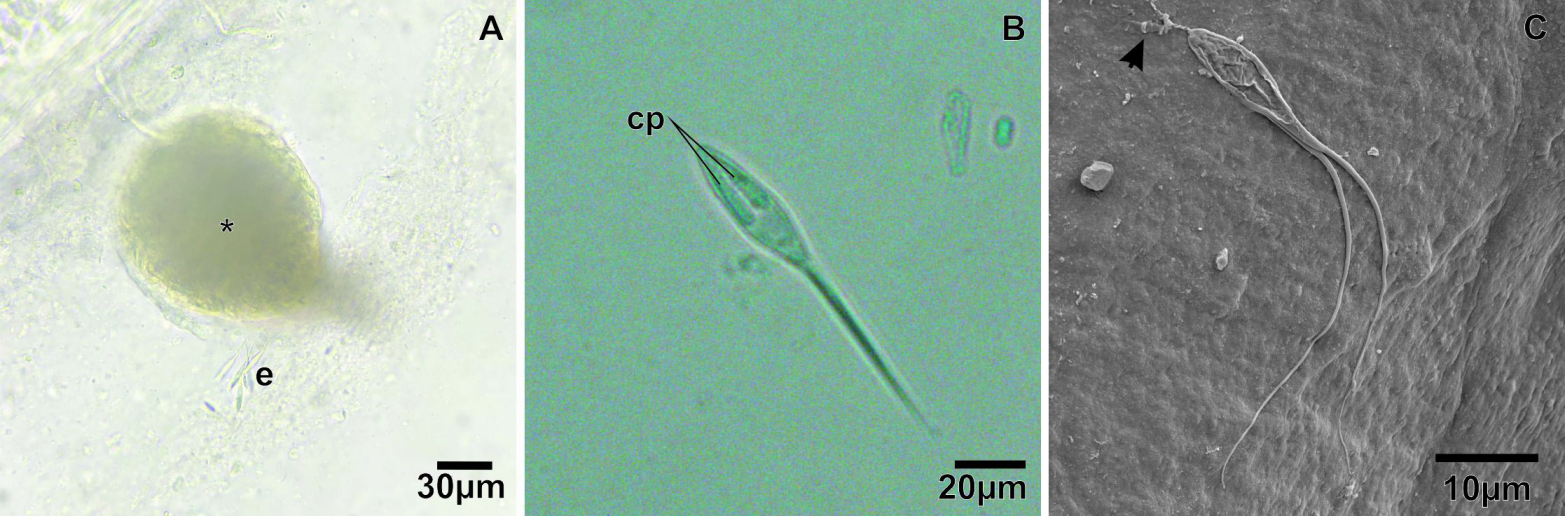
Light microscopy and scanning electron microscopy (SEM) photomicrographs of *Henneguya* sp. in the gill filament of *Pygocentrus nattereri.* **A** - Cyst (*) and myxospores (e) of *Henneguya* sp. **B** - Mature myxospore of *Henneguya* with two polar capsules (cp). **C**- SEM of myxospore of *Henneguya* sp. with highlight to the extruded nematocysts (arrow).

**Figure 3:**
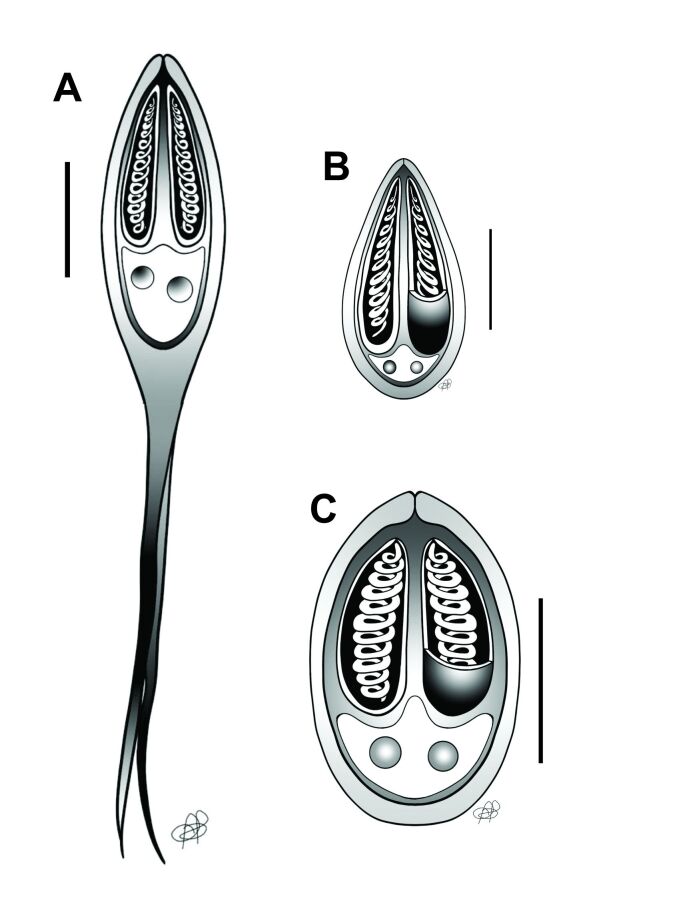
Illustrative drawings of mature myxospores of *Henneguya* sp. and *Myxobolus* sp1. and *Myxobolus* sp. 2 of *Pygocentrus nattereri*. **A** - Myxospore of *Henneguya* sp. **B** - Mature myxospores of *Myxobolus* sp. 2. **C** - Mature myxospores of *Myxobolus* sp. 1. Scale bar: 5 µm.

The *P. nattereri* specimens measured 22.2 ± 1.4 cm in total length and 351.8 ± 102 g in body weight. Rounded cysts (Figure 2A) were observed in the gill filaments containing myxospores that had an ellipsoidal shape and caudal projections (Figure 2B-C; Figure 3A), measuring 32.4 µm in total size, 12.9 µm in myxospore body length, 4.5 µm in width, and had two polar capsules measuring 6.6 µm in length by 1.1 µm in width, with 14 to 15 turns of the polar filament. These myxospores presented a morphology related to the genus *Henneguya* and with a percentage of infected animals of 44% (8/18).

**Figure 4:**
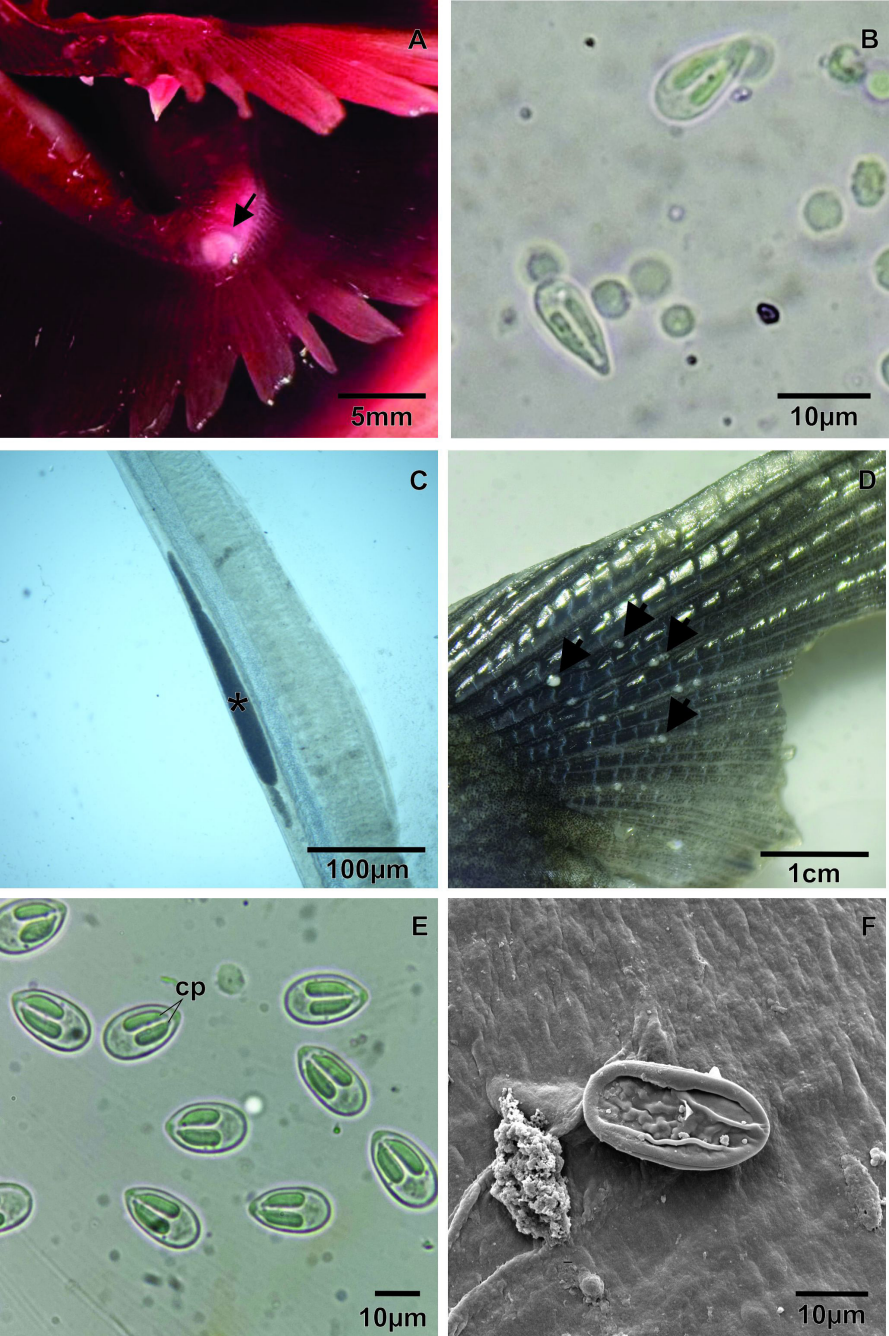
Macro- and light-microscopy and scanning electron microscopy micrographs of *Myxobolus* sp. in *Pygocentrus nattereri*. **A** - Rounded cyst of *Myxobolus* sp. 2 in the gill arch (arrowhead). **B** - Mature myxospores of *Myxobolus* sp. 2 with teardrop-shaped morphotype found in the gill arch and kidney. **C** - Elongated cyst (*) in the gill filament. **D** - Several cysts in the caudal fin (arrowhead). **E** - Mature myxospores of *Myxobolus* sp. 1 with oval morphotype, found in the gill filament, caudal fin, and kidney, with a highlight of the two polar capsules (cp). **F** - Scanning electron microscopy micrographs of *Myxobolus* sp1. myxospore.

Rounded cysts were also observed in the gill arch (Figure 4A) and caudal fin (Figure 4D), whereas elongated cysts were observed in the gill filaments (Figure 4C). Mature myxospores were present in the caudal kidney, and morphometric analyses revealed two morphotypes of *Myxobolus* myxospores: one with an oval shape, *Myxobolus* sp1. (oval) (Figure 4E; Figure 3C), located in the gill filament, caudal fin, and kidney, and the other more elongated, drop-shaped *Myxobolus* sp2. (drop) (Figure 4B; Figure 3B), present in the gill arch and caudal kidney (Table 2). Of the hosts analyzed, 22% (4/18) were parasitized by *Myxobolus* myxospores.

**Figure 5:**
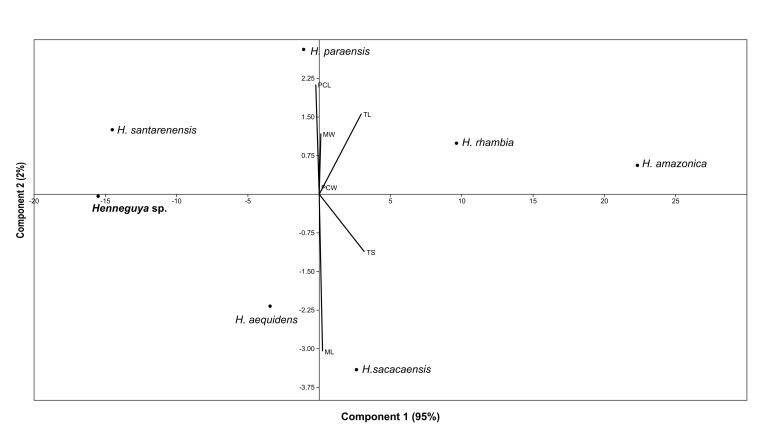
Principal component analysis of morphometric data of *Henneguya* sp. TS: total size; TL: tail length; ML: myxospore length; MW: myxospore width; PCL: polar capsule length; PCW: polar capsule width.

**Figure 6:**
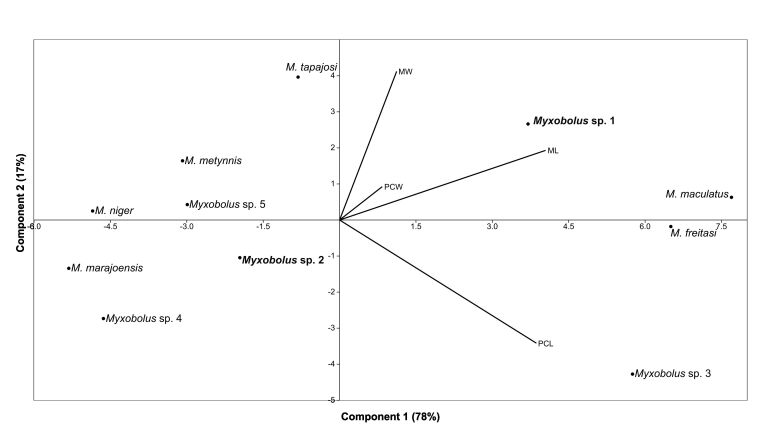
Principal component analysis of morphometric data of *Myxobolus* sp. ML: myxospore length; MW: myxospore width; PCL: polar capsule length; PCW: polar capsule width.

Axes 1 and 2 of the PCA represented 97% of the variance of the morphometric data of *Henneguya* sp., with axis 1 (95%) being influenced mainly by tail length and total size, while axis 2 (2%) was spore length and width and polar capsule length (Figure 5). While in relation to the PCA of the morphometry of *Myxobolus* sp. the total variance was 95%, axis 1 represented by the variables spore length and polar capsule length represented 78%, while axis 2 with 17% of variance was influenced by spore width and capsule length (Figure 6).

## Discussion

*Henneguya* species frequently parasitize Amazonian fish (Rocha et al., 1992; Azevedo & Matos, 1995; Casal et al., 2003; Videira et al., 2015; Velasco et al., 2016; Ferreira et al., 2020; Capodifoglio et al., 2020). However, few species have been described parasitizing piranhas, namely *Henneguya striolata* Casal, Matos & Azevedo, 1997 (Casal et al., 1997) parasitizing gill lamellae of *Serrasalmus striolatus* Steindachner, 1908, in the Amazon River (Casal et al., 1997); *Henneguya pilosa* Azevedo, Matos, 2003, from the gills of *Serrasalmus altuvei* Ramírez, 1965 in Teresina, state of Piauí, Brazil; *Henneguya curvata* Barassa, Adriano, Arana, Cordeiro 2003 from the gills of *Serrasalmus spilopleura* Kner, 1858 from Rio das Pedras, Campinas, state of São Paulo, Brazil.

*Henneguya* species are preferentially found in the gills of host fish species (Matos et al., 2005; Velasco et al., 2016); however, they have also been found in the gallbladder, tegument, fins, and kidneys (Azevedo & Matos, 1996; Eiras et al., 2004; Azevedo et al., 2008; Müller et al., 2023). In this study, the occurrence of *Henneguya* sp. parasitizing the gill filaments of *P. nattereri* was recorded at a low prevalence (44.0%). However, higher prevalence values ​​were recorded for *Henneguya* sp*.* in *Satanoperca jurupari* Heckel, 1840 (Ferreira et al., 2020) at 57.1% and *Cichla temensis* Humboldt, 1821 (Velasco et al., 2016) at 60.0%. In addition, Naldoni et al. (2018) reported a prevalence of 100% for *Henneguya santarenensis* Naldoni, Maia, Correia, Silva & Adriano, 2018, in *Phractocephalus hemioliopterus* Bloch & Schneider, 1801.

Morphometric analyses showed that *Henneguya* sp. of *P. nattereri* differs from the compared species. However, little similarity was observed in total myxospore size with *H. santarenensis,* found in the gills of *P. hemioliopterus* in state of Pará, Brazil (Naldoni et al., 2018).

Parasites of the genus *Myxobolus* are common in freshwater and saltwater fish in both natural and farmed environments (Capodifoglio et al., 2016, 2019). However, in this study, the prevalence values ​​were lower than those found in other freshwater fish, *Myxobolus tapajosi* Zatti, Atkinson, Maia, Corrêa, Bartholomew and Adriano, 2018, in *Brachyplatystoma rousseauxii* Castelnau, 1855 at 23.5% (Zatti et al., 2018), *Myxobolus metynnis* Casal, Matos & Azevedo, 2006 in *Metynnis argenteus* Ahl, 1923 at 26.0% (Casal et al., 2006), *Myxobolus* sp. in *Metynnis hypsauchen* Muller & Troschel, 1844 at 60.0% (Oliveira et al., 2020), and *Myxobolus freitasi* (Sindeaux-Neto, Velasco, Silva, Matos, Silva, Gonçalves & Matos, 2021 in *Brachyhypopomus beebei* Schultz, 1944 at 60.0% (Sindeaux-Neto et al., 2021).

*Myxobolus* species infect different fish organs, including the gills, kidneys, heart, skin, nervous system, and urinary bladder (Casal et al., 2002; Eiras et al., 2005; Maciel et al., 2011; Sindeaux-Neto et al., 2021; Silva et al., 2023; Velasco et al., 2024). In *P. nattereri*, *Myxobolus* sp. was reported in only two sites of infection, *Myxobolus dermatoulcerans* Stilwell, Stilwell, Camus, Ware, Rosser & Griffin 2020 in the skin (Stilwell et al., 2020) and *Myxobolus colossomatis* Molnar & Békesi, 1993, in the circulating blood (Úngari et al., 2022). In the present study, *Myxobolus* sp. were found in the kidneys, gill arch, and caudal fins. These same sites were also infected by *Myxobolus* sp. in other fish species of the Serrasalmidae family, such as *Metynnis maculatus* Kner, 1858, *Metynnis hypsauchen* Muller & Troschel, 1844 (Oliveira et al., 2020), and *Metynnis lippincottianus* Cope, 1870 (Façanha et al., 2024), which are parasitized by *Myxobolus* sp. in the kidney. In *Colossoma macropomum* Cuvier, 1816, *Myxobolus colossomatis* Molnár & Bekesi, 1993 was found in the fins, arches, and gill filaments of hosts (Müller et al., 2013).

Two morphotypes of *Myxobolus* were found in the kidneys of *P. nattereri*: *Myxobolus* sp1. (oval) and *Myxobolus* sp.2 (drop/pyriform). Similar morphology to *Myxobolus* sp.1 was reported in *C. macropomum* for *M. colossomatis* infecting the liver, spleen, pyloric cecum, fin, intestinal wall, digestive tract, and branchial arch of hosts (Capodifoglio et al., 2019), in *M. lippincottianus* for *Myxobolus* sp. in the blood and kidney (Façanha et al., 2024), and in *Metynnis argenteus* Ahl, 1923, the subcutaneous connective tissue of the orbicularis region parasitized by *Myxobolus metynnis* Casal, Matos & Azevedo (Casal et al., 2006). The morphology of *Myxobolus* sp. 2 resembles the myxospore of *M. maculatus* described in the liver of *M. maculatus* (Casal et al., 2002) and of *Myxobolus* sp. in *M. lippincottianus* (Façanha et al., 2024).

Myxozoa can develop in several internal organs and spread through the bloodstream to other organs until they reach the kidney, where they can develop or be used as a deposit, where myxospores from the bloodstream are collected, stored, and destroyed by macrophages in the interstitium (Molnár, 2007). As an excretory organ, the caudal kidney serves as a passage for the release of myxospores into the environment through the host's urine (Manrique et al., 2017).

In the present study, morphometrically, *Myxobolus* sp.1 and *Myxobolus* sp.2 were different from those of all the species compared. However, *Myxobolus* sp1. resemble the myxozoan *Myxobolus maculatus* Casal, Matos & Azevedo, 2002, found in the kidneys of *M. maculatus* (Casal et al., 2002) based on myxospore length. However, analysis of its morphology, which presents an oval shape, *Myxobolus* sp1. was similar to that *Myxobolus* sp. collected from the blood and kidneys of *M. lippincottianus* (Façanha et al., 2024). The morphometric analyses of *Myxobolus* sp.2 revealed similarities with *Myxobolus* sp.4 reported in the blood and kidney of *M. lippincottianus* based on myxospore length, width, and length of the polar capsules. However, its drop-shaped morphology resembled that of *M. maculatus* (Casal et al., 2002).

This is the first study to record the presence of *Henneguya* sp. in the gill filament, and *Myxobolus* sp. in the gill arch, gill filaments, caudal fin, and caudal kidney, highlighting the presence of two morphotypes parasitizing the kidneys of *P. nattereri* from the municipality of Pracuúba in the state of Amapá.
